# Central nervous system uptake of intranasal glutathione in Parkinson’s disease

**DOI:** 10.1038/npjparkd.2016.2

**Published:** 2016-02-25

**Authors:** Laurie K Mischley, Kevin E Conley, Eric G Shankland, Terrance J Kavanagh, Michael E Rosenfeld, John E Duda, Collin C White, Timothy K Wilbur, Prysilla U De La Torre, Jeannie M Padowski

**Affiliations:** 1 Department of Radiology, University of Washington (UW), Seattle, WA, USA; 2 Graduate Program in Nutritional Sciences, School of Public Health, University of Washington, Seattle, WA, USA; 3 School of Naturopathic Medicine, Bastyr University Research Institute, Kenmore, WA, USA; 4 Department of Environmental & Occupational Health Sciences, School of Public Health, University of Washington, Seattle, WA, USA; 5 Michael J. Crescenz VA Medical Center, Philadelphia, PA, USA; 6 Department of Neurology, Perelman School of Medicine at the University of Pennsylvania, Philadelphia, PA, USA; 7 Department of Biomedical Sciences, Elson S. Floyd College of Medicine, Washington State University, Spokane, WA, USA; 8 Department of Experimental and Systems Pharmacology, College of Pharmacy, Washington State University, Spokane, WA, USA

## Abstract

Glutathione (GSH) is depleted early in the course of Parkinson’s disease (PD), and deficiency has been shown to perpetuate oxidative stress, mitochondrial dysfunction, impaired autophagy, and cell death. GSH repletion has been proposed as a therapeutic intervention. The objective of this study was to evaluate whether intranasally administered reduced GSH, (in)GSH, is capable of augmenting central nervous system GSH concentrations, as determined by magnetic resonance spectroscopy in 15 participants with mid-stage PD. After baseline GSH measurement, 200 mg (in)GSH was self-administered inside the scanner without repositioning, then serial GSH levels were obtained over ~1 h. Statistical significance was determined by one-way repeated measures analysis of variance. Overall, (in)GSH increased brain GSH relative to baseline (*P*<0.001). There was no increase in GSH 8 min after administration, although it was significantly higher than baseline at all of the remaining time points (*P*<0.01). This study is the first to demonstrate that intranasal administration of GSH elevates brain GSH levels. This increase persists at least 1 h in subjects with PD. Further dose–response and steady-state administration studies will be required to optimize the dosing schedule for future trials to evaluate therapeutic efficacy.

## Introduction

Glutathione (GSH) deficiency is one of the earliest biochemical perturbations in Parkinson’s disease (PD),^1,2^ leading to the hypothesis that GSH supplementation may have therapeutic value in alleviating PD symptoms or modifying progression.^3^ Reduced GSH (GSH; γ-L-glutamyl-L-cysteinylglycine) is a tripeptide involved in the scavenging of hydroxyl radical (*OH) and singlet oxygen, the reduction of H_2_O_2_, and for cellular detoxification through GSH-*S*-transferases.^4,5^ Deficient GSH synthesis has been associated with oxidative stress in aging,^6^ and GSH concentrations decrease with age, a factor thought to explain, in part, why the elderly are at greater risk of neurodegenerative diseases.^7,8^

Two major factors have limited progress toward investigating the utility of GSH supplementation as a therapeutic strategy in PD. First, GSH bioavailability is very low following oral administration. Alternative repletion strategies have focused on oral administration of GSH precursors (e.g., cysteine and glycine supplementation^9^), and intravenous administration of GSH,^10^ which although promising, is invasive and inconvenient, and therefore unlikely to be a practical solution. Second, the inability to quantify central nervous system (CNS) GSH concentrations *in vivo* has substantially hindered therapeutic trials targeting CNS augmentation. The current study addressed these limitations by testing a noninvasive nasal GSH repletion strategy, and measuring CNS uptake via proton magnetic resonance spectroscopy (^1^H-MRS).

^1^H-MRS is a noninvasive approach that enables the determination of *in vivo* concentrations of specific neurochemicals, including GSH. GSH brain concentrations are not commonly measured using ^1^H-MRS, because relative to other ^1^H-MRS-detectable neurochemicals (e.g., creatine (Cr), choline, *N*-acetylasparate), GSH concentrations are substantially lower. In addition, the GSH signal from ^1^H protons of the cysteinyl β-CH_2_, which forms a resonance peak at 2.95 p.p.m., is obscured by nearby spectral peaks from other neurochemicals. The development of editing techniques such as Meshcher-Garwood point resolved spectroscopy (MEGA-PRESS) to effectively suppress nearby resonance peaks of other neurochemicals (e.g., Cr: 3.03 p.p.m.; aspartate: 2.82 p.p.m.; GABA: 3.01 p.p.m.) has provided a practical method for the measurement of GSH concentrations by ^1^H-MRS.^11^ MEGA-PRESS editing has been used to demonstrate alterations in GSH brain concentrations in a handful of conditions, including normal aging,^12^ Alzheimer’s disease,^13^ and schizophrenia.^14^

Little is known about the capacity of exogenously administered GSH, or its precursors, to modify CNS GSH levels. In a 2013 study,^15^ the GSH precursor *N*-acetyl cysteine (NAC) was administered as a single 60-min intravenous infusion (150 mg/kg) to individuals with PD and healthy controls. NAC administration increased brain GSH concentrations by 55% in subjects with PD, and 34% in healthy controls (*n*=3). The authors reported that maximal brain GSH concentrations were measured ~90–110 min after the start of the infusion, and had not returned to baseline levels by 120 min after the start of the infusion.^15^ These results support the hypothesis that NAC is capable of crossing the blood–brain barrier and providing cysteine substrate to CNS cells, thus enhancing GSH synthesis. Although these findings appear promising, the utility of intravenous NAC repletion as a therapeutic strategy in PD is greatly limited by invasiveness and inconvenience of intravenous delivery.

Recently, a dose-dependent increase in cerebrospinal fluid (CSF) total and reduced NAC concentrations was demonstrated in association with oral NAC administration.^16^ Despite increases in NAC concentrations, there was no observed increase in CSF total or reduced GSH concentrations, presumably because the conversion of NAC to GSH occurs intracellularly. Under normal physiologic conditions, intracellular GSH concentrations of neurons are 250- to 500-fold higher than in the CSF,^17^ thus limiting the utility of CSF to serve as a biomarker of cellular GSH status. In spite of their limitations, these studies are the first to demonstrate a capacity of exogenously administered NAC to reach the CNS. An oral NAC supplementation trial is underway to assess changes in brain GSH levels using similar ^1^H-MRS.^18^

Intranasal administration of reduced GSH, (in)GSH, could be an effective approach for delivery of GSH to the CNS. Many studies suggest that small, polar molecules may be able to “bypass” the blood–brain barrier with nasal delivery, as the interface between the nasal cavity and brain is considered a potential point of vulnerability in the blood–brain barrier. On the basis of the biological plausibility and anecdotal case reports of clinical improvement, (in)GSH has been recommended as an off-label therapy for GSH augmentation in PD since 2004.^19–21^ Recently, a double-blind, placebo-controlled randomized clinical trial of phase I study of (in)GSH in PD (*n*=30) demonstrated (in)GSH was safe and tolerable, and both active study arms demonstrated an improvement over placebo in total Unified PD Rating Scale scores, specifically in activities of daily living and motor Unified PD Rating Scale subscores.^22^

The current proof-of-concept study was designed to evaluate whether (in)GSH is capable of augmenting CNS GSH concentrations, as measured by ^1^H-MRS.

## Results

### Subject screening and enrollment

**Figure 1:**
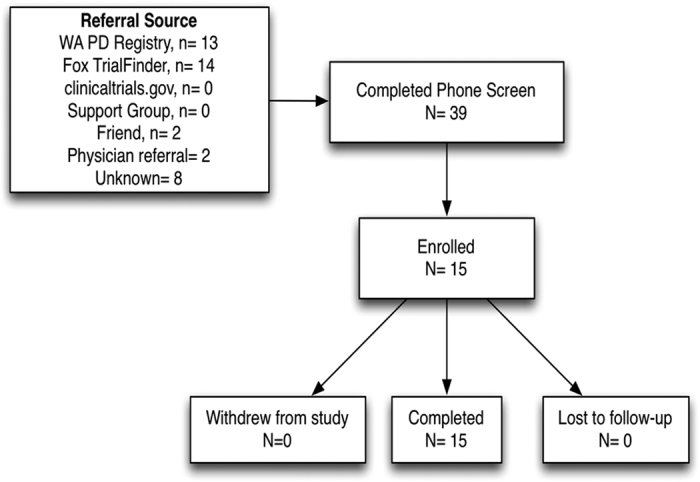
CONSORT enrollment algorithm.

In all, 31 individuals were screened in order to identify 15 who qualified. Most study referrals came from the Michael J Fox Foundation Trial Finder (45%) and Washington State PD Registry (42%), with health-care providers and friends contributing to the remaining referrals (13%). The subject population was highly diverse in terms of age, socioeconomic status, education, and geographic neighborhoods throughout the Pacific Northwest, although all participants were Caucasian. The characteristics of study participants are presented in Table 1 and the enrollment algorithm is presented in Figure 1.

### Study medication quality and tolerability

Independent analysis of three separate samples compounded to contain 200 mg/ml demonstrated the product potency to be 190 mg/ml (95%) upon receipt, and potency reduced to 144 mg/ml (72%) at 4 weeks in one sample, and 161 mg/ml (80.5%) at 6 weeks in a separate batch.

One participant experienced a single adverse reaction to the study medication, cephalgic paresthesia, which resolved within 1 h.

### Changes in brain GSH levels after (in)GSH administration

The duration of post-dose measurements was driven by participant comfort and scheduled time in scanner, up to the duration of time approved by the institutional review board. Six subjects underwent three post-dose measurements, eight subjects underwent four post-dose measurements, and one subject underwent a fifth measurement (Table 2).

**Figure 2:**
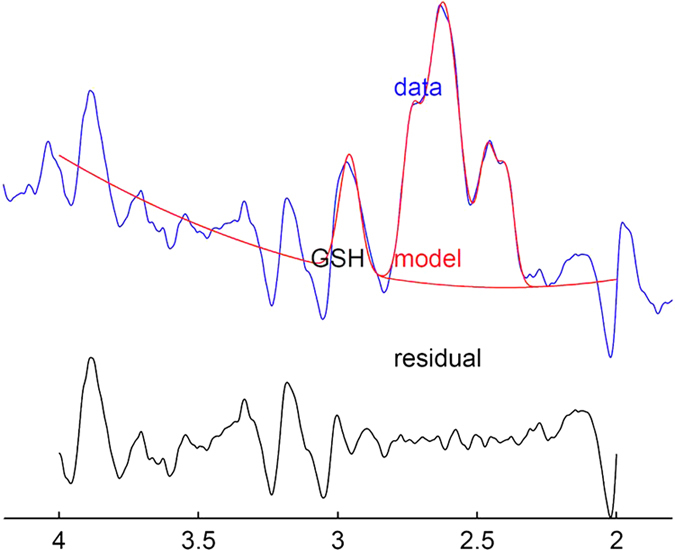
Representative fit to glutathione (GSH) peak. The GSH-edited spectrum is shown in blue. The upper red line illustrates the best fit of a 5-Gaussian model to GSH and co-edited molecules (overall fit), and the lower red line illustrates the fit of a simple Gaussian model to the GSH peak (GSH quantification). Below the plot, the residual between the spectrum and model best fit is shown in black.

GSH-edited spectra were successfully obtained from all 15 subjects (representative spectrum, Figure 2). For one subject, the spectrum obtained at the second post-dose time point was of insufficient quality, and was omitted from analysis. For six subjects, baseline GSH peaks were undetectable. For these six subjects, baseline GSH levels were all assigned the same value (the lowest measured GSH/Cr ratio value across all subjects and scans, divided by 2), in order to calculate the absolute change in GSH/Cr ratio relative to baseline. Thus, of the 70 spectra acquired, a total of 7 spectra were omitted from analysis. For the remaining 63 spectra, mean fit error was 38%. The combined fit error for GSH and Cr varied by less than a factor of 2 over the course of each subject’s serial scans. For point-resolved spectroscopy (PRESS) spectra, all scans met the stated quality control criteria. CSF fraction within the voxel ranged from 7 to 25% (mean 17%, ±4.9% s.d.).

**Figure 3:**
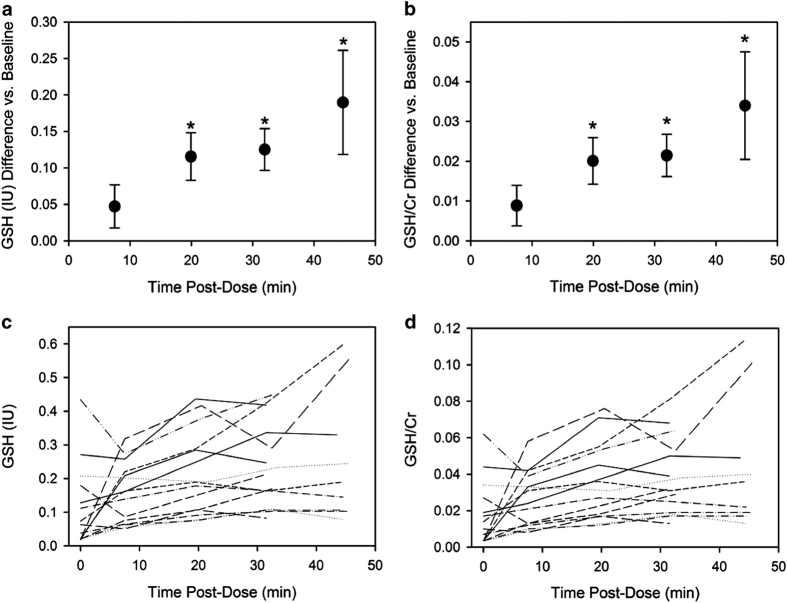
Differences in glutathione (GSH) levels, as absolute GSH (**a**) and GSH/Cr (**b**), relative to baseline versus time after 200 mg intranasally administered GSH. Data are presented as mean±s.e.m. Asterisks indicate points that are significantly different from baseline (one-way repeated measures analysis of variance comparing each point to baseline, *P*<0.05 after correction for multiple comparisons). Shown below are time courses of change in absolute GSH (**c**) and GSH/Cr (**d**) in individual subjects over time post dose.

Mean GSH levels increased consistently with time relative to baseline (Table 2 and Figure 3a,b), although levels fluctuated somewhat among individual subjects (Figure 3c,d). GSH/Cr (as well as absolute GSH levels) were significantly different from each other (one-way repeated measures analysis of variance, *P*<0.001). The increase in GSH/Cr or absolute GSH immediately after (in)GSH administration (7.5 min) was not significantly different from baseline, however, GSH levels were significantly higher than baseline at all of the remaining time points (*P*<0.05; Figure 3a,b). Between the baseline and the 45-min scan, there was a mean 269% increase in GSH/Cr (240% increase in absolute GSH).

## Discussion

To our knowledge, this is the first study to demonstrate an increase in CNS GSH levels with a noninvasive GSH augmentation strategy. GSH augmentation as a potential therapeutic strategy in CNS disease has been suggested for decades,^23^ although repletion efforts have been hindered by inability to assess human CNS GSH concentrations *in vivo* and poor oral absorption of GSH.^24^ Here we demonstrate that both of these obstacles are surmountable by using a ^1^H-MRS editing method to measure CNS GSH levels, and a noninvasive (in)GSH administration strategy. GSH augmentation deserves investigation as a beneficial therapeutic approach for not only PD but also numerous other CNS disorders for which GSH deficiency and GSH-related enzyme deficits have been documented (multiple sclerosis,^25,26^ autism,^27–29^ Alzheimer’s disease,^30,31^ schizophrenia,^32,33^ and bipolar disease^34^).

Previously, i.v. NAC was demonstrated by magnetic resonance spectroscopy (MRS) to augment CNS GSH concentrations.^15^ Although effective, i.v. therapy requires trained medical personnel for administration, thus raising costs, patient burden for clinic visits, and risk of discomfort and phlebitis. Ours is the first study to demonstrate CNS GSH augmentation using a noninvasive, self-administered therapy in humans. The single dose, short (1 h) observation period, and lack of placebo arm are limitations in this proof-of-concept study and provide direction for follow-up studies.

GSH levels were calculated both relative to Cr (GSH/Cr peak area ratio) and as absolute (water-referenced) GSH concentrations. Although water-referenced neurochemical concentrations are considered by some as the “gold standard” approach for MRS, there are numerous technical challenges and assumptions that can limit the utility of water-referenced measurements.^35^ Alternatively, reporting of neurochemical levels relative to a reference neurochemical in the same voxel is a common approach, as measurements are technologically uncomplicated (only a single spectrum must be collected) and no correction for partial-volume effects is required. However, a ratio approach can complicate interpretation of data, if it is unclear whether the reference neurochemical is altered by treatment as well. For this reason, both GSH/Cr ratios and absolute GSH concentrations are reported. We observed good correspondence between the relative and absolute GSH levels (Figure 3), and the statistical significance of GSH level changes with time post dose was the same regardless of the quantitation approach. In addition, although comparison of absolute MRS neurochemical concentrations across different instruments and sites is highly challenging, the baseline absolute GSH levels that we observed (mean 0.109 IU (institutional units); range 0.0183–0.435) are within the ranges reported in the literature for postmortem CNS concentrations of GSH in subjects with PD.^2^ Of note, the range of reported brain GSH concentrations is extremely variable, ranging over an order of magnitude.^15,36^ The strength of this study lies in the demonstration of a consistent increase in brain GSH levels with time post dose. Absolute Cr concentration in the voxel (mean 6.09 IU) were also comparable to reported values.^37^

It should be noted that this study was not designed to differentiate between GSH in brain tissue versus CSF. However, as reported concentrations of GSH are in the range of 0.2 μmol/l in CSF and 1 mmol/l in brain tissue,^7^ GSH in CSF would not appreciably change the MRS determination of GSH in brain tissue. Owing to the velocity of blood movement through the voxel, MRS does not detect GSH signal from blood, thus, the measured GSH values reflect only GSH in brain parenchyma or CSF.

This pilot study was designed to demonstrate that (in)GSH results in an increase in the GSH signal in brain, and not to generate a comprehensive pharmacokinetic profile of the increased brain GSH signal. In light of the data generated in this study, a more complete pharmacokinetic investigation designed to quantify the magnitude and duration of increase in GSH is warranted. Additional studies could be directed toward optimizing delivery techniques, dosing schedules, product stability, and intranasal formulations.

Recently, a small phase I/IIa study of (in)GSH in PD demonstrated a mild symptomatic improvement in PD symptoms as measured by the Unified PD Rating Scale, with return of symptoms upon withdrawal of (in)GSH.^38^ Although numerous questions remain about the mechanism by which GSH may ameliorate PD symptoms, this study demonstrates that a single dose of (in)GSH does, in fact, reach the target tissue. In addition to biological activity as an essential intracellular antioxidant, GSH facilitates the clearance of metabolic waste via GSH-*S*-transferases and may function as a neuropeptide.^39^ In astrocytes, GSH serves as a reservoir for cysteine, glycine, and glutamic acid, each with their own biochemical activities. Glycine, a *N*-methyl-D-aspartate receptor agonist, has been shown to significantly improve negative symptoms in patients with schizophrenia when supplemented.^40^ Cysteine availability has been shown to regulate extracellular glutamate concentrations, and thus neuronal excitability, via the cystine–glutamate antiporter.^41^ Using 123-IFP-CIT single-photon emission computed tomography, high doses of (iv)GSH significantly influenced putamen dopamine transporter in PD patients.^42^ A follow-up study is underway to evaluate the effects of 3 months of (in)GSH on PD symptom status and MRS GSH concentrations. Considering the potential for therapeutic development of (in)GSH, the established safety and tolerability data, biological plausibility, and pilot level clinical evidence of benefit are further supported with this demonstration that (in)GSH is able to augment brain GSH levels.

## Materials and methods

Institutional Review Board approval was obtained at the University of Washington for this single-center study of 15 participants with mid-stage PD. Recruitment occurred through the Michael J. Fox Foundation Trial Finder,^43^ the Washington PD Registry,^44^ ClinicalTrials.gov (NCT02324426), and referral from local health-care providers. Inclusion criteria required participants to be over age 18, read and speak English, have a Hoehn & Yahr score between 2 and 3 (bilateral disease, not severely disabled), and have three or more of the required positive criteria for the diagnosis of definite PD from Step 3 of the UK Brain Bank Diagnostic Criteria for PD.^45^ Exclusion criteria included any contraindication to MRI, history of epilepsy, stroke, brain surgery, or structural brain disease, pregnancy, history of sulfur sensitivity, ongoing asthma or drug dependence, ongoing chronic diseases, history of mental illness, or acute infection during the prior 30 days. Informed consent was obtained from all participants. In light of evidence from animal models that brain GSH concentrations peak in the morning,^46^ the single study visit was scheduled at 0700 hours for each participant in an attempt to control for circadian fluctuations.

### Brain GSH assessment

#### Imaging and voxel selection

**Figure 4:**
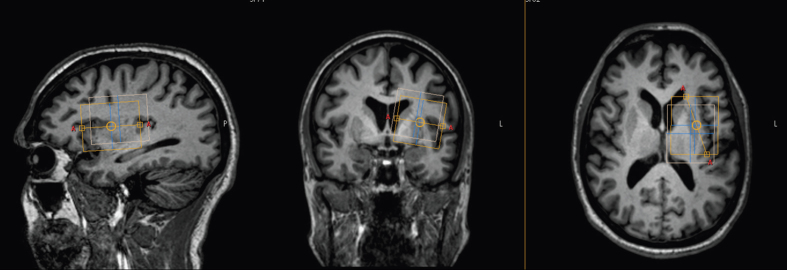
Volume of interest. For all participants, glutathione (GSH) was administered into the left nostril, and a voxel was placed over a 4×4×5-cm region centered on the left dorsal putamen at the level of the lentiform nucleus.

Brain GSH levels were determined using a Philips Achieva 3.0-T whole-body scanner (Best, The Netherlands) equipped with a 32-channel SENSE phased-array head coil. From each subject, a detailed brain image was first acquired, using a magnetization-prepared rapid gradient echo^47^ high-resolution T1-weighted sequence (repetition time=6.6 ms, echo time=3.0 ms, flip angle=8°, matrix=256×240, slices =170, and slice thickness=1 mm). Images were evaluated in real-time to select a cubic volume of interest, 4×4×5 cm, centered over the left dorsal putamen at the level of the lentiform nucleus. As CNS GSH concentrations are thought to be reduced in PD, a relatively large voxel size was selected in order to maximize signal to noise. The dorsal putamen was selected as the center of the volume of interest due to its relatively homogenous mix of neurons and astrocytes, and suitable distance from bone and other regions that could compromise signal quality. The voxel was positioned to avoid the skull and, to the extent possible, the left lateral ventricle (Figure 4).

#### ^1^H-magnetic resonance spectroscopy

The cysteinyl β-CH_2_ of GSH exhibits a characteristic chemical shift at 2.95 p.p.m., which distinguishes it from other cysteine-based molecules.^48^ GSH levels were determined within the volume of interest using MEGA-PRESS double-editing for the cysteinyl β-CH_2_ residue of GSH^7^ (repetition time=2,000 ms, echo time=122 ms, free induction decay points=2,048, spectral width=2,000 Hz, number of averages=8 per phase cycle ON or OFF, 320 acquisitions total requiring just under 11 min). Spectral editing was accomplished by refocusing GSH J-evolution during every other acquisition (ON), using a 43-ms Gaussian pulse centered at the cysteinyl α-CH resonance of GSH at 4.56 p.p.m. During the alternate acquisitions (OFF), the pulse was applied symmetrically about the water peak. The difference-edited GSH spectrum was generated by subtraction of the OFF and ON spectra.

To facilitate quantification of GSH, additional spectra were collected from the same volume of interest (Figure 4) using a short-echo PRESS sequence with vapor water suppression (repetition time=2,000 ms, echo time=36 ms; free induction decay points= 2,048, spectral width=2,000 Hz, number of averages=64). To account for T_2_-weighting differences, PRESS water spectra were also collected using both echo times (echo time=122 or 68 ms, repetition time=2,000 ms, free induction decay points=2,048, spectral width=2,000 Hz, number of averages=8).

After baseline MEGA-PRESS and PRESS spectra were acquired, 200 mg GSH was self-administered into the left nostril by each subject inside the scanner without repositioning. Immediately after administration (within 2 min), serial GSH MEGA-PRESS spectra were obtained over the course of up to 62 min post dose (11 min per scan, for a total of three to five measurements post dose). For consistency, the study medication was always administered in the left nostril and spectra were collected ipsilaterally.

#### Quantification

For each subject, brain GSH levels were quantified from difference-edited spectra using the Gannet 2.0 Toolkit, a Matlab-based automated program for analyzing MEGA-PRESS spectra.^49^ Gannet processing steps include 3 Hz exponential line broadening, and frequency- and phase-correction of individual spectra. The edited spectra are fit with Gaussian models, and the GSH signal is expressed relative to the Cr signal; GSH/Cr ratio. Assessment of inter-and intra-subject data quality was accomplished by comparing fit errors (calculated as the s.d. of the residual of the analyte peak, expressed as a percentage of the analyte peak amplitude). In cases where the GSH peak was undetectable, a value (the lowest measured GSH/Cr ratio value across all subjects and scans, divided by 2) was assigned.

To calculate absolute (i.e., water-referenced) GSH levels from GSH/Cr ratios, concentrations of total Cr (Cr plus phosphocreatine) were calculated. Cr concentrations were determined from PRESS spectra using standard model-fitting procedures (LCModel software version 6.2-0T (ref. 50)). A decomposition-fitting algorithm was used to subtract residual water signals. Free induction decays were zero-filled, smoothed with a 1.1-Hz exponential-dampening filter, and then zero- and first-order phase corrected. Quality control criteria included a peak width ⩽0.1 p.p.m., signal-to-noise ratio ⩾5, and Cramer–Rao lower bounds <20% (as percentage of the estimated concentration). Absolute (water-referenced) Cr concentrations were determined by scaling the spectrum to the unsuppressed water peak, resulting in values with IU that approximate millimolar (mmol/l) concentrations.

To correct for the partial-volume effect, fractions of CSF and brain tissue (gray and white matters) were determined within the voxel using FSL FAST segmentation.^51^ As GSH is known to be present in CSF at very low concentrations (~0.2 μmol/l in both healthy and PD subjects),^52^ relative to brain tissue concentrations (~1 mmol/l),^7^ LCModel-calculated Cr concentrations (*C*_measured_) were corrected (*C*_corrected_) using the following formula, which assumes negligible contribution of CSF GSH to the total GSH signal:

$$C_{\mathrm{corrected}}=C_{\mathrm{measured}}-\left(\frac{1}{\mathrm{fraction}\;\mathrm{CSF}\;\mathrm{in}\;\mathrm{voxel}}\right)$$

Absolute GSH levels (IU) were calculated by multiplying GSH/Cr ratios by CSF-corrected absolute Cr concentrations (IU).

Changes in GSH levels with time post dose were calculated as the difference in GSH/Cr peak ratios for each subject at each time point (GSH/Cr_post dose_−GSH/Cr_baseline_), or similarly, the difference in absolute GSH for each subject at each time point.

### Study medication

Powdered GSH was obtained from MEDISCA (Plattsbergh, New York, USA) and compounded by Key Pharmacy (Federal Way, WA, USA). The study medication was stored in a study refrigerator and protected from light until 30 min before administration, when it was allowed to come to room temperature, for participant comfort during administration. All participants were administered an identical intervention (1cm^3^ of saline containing 200 mg GSH) using a syringe attached to a Mucosal Atomization Device supplied by Wolfe-Tory Medical (Salt Lake City, UT, USA). This dose is the highest dose meeting tolerability and safety criteria in the phase I study of (in)GSH in PD.^38^ As a quality control measure, medication samples were sent for independent potency analysis (Eagle Analytical, Houston, TX, USA) upon receipt, and at 4 and 6 weeks after production.

### Statistical analysis

Using data generated from a pilot study and G*Power 3.1 software (Düsseldorf, Germany), it was determined that a sample size of 15 would provide 80% power to detect an increase in CNS GSH concentrations between pre- and post-administration values, with an accepted alpha value of 0.2. Descriptive statistics for study participants are listed in Table 1.

A single brain GSH level was determined from each 11-min MEGA-PRESS acquisition. For the purpose of illustrating changes in GSH level with time, levels were treated as corresponding to the midpoint of each scan. Changes in brain GSH levels over time were determined as the difference between the GSH/Cr ratio (or absolute GSH) at each time point, relative to baseline. Significance was determined by one-way repeated measures analysis of variance, and the Holm–Sidak method for multiple comparisons versus the control group, using SigmaPlot 10.0 software (Systat Software, San Jose, CA, USA). Variance among all groups was not statistically different.

## Figures and Tables

**Table 1:** Characteristics of study participants

	*Total (*n=*15)*	*Min*	*Max*
*Gender*			
Male	11 (73%)		
Female	4 (27%)		

*Age (years)*			
Mean (s.d.)	65.5 (11.2)	44	83
Race: Caucasian	15 (100%)		
Years since PD diagnosis (s.d.)	6.1 (6.2)	0.7	23
			
*UPDRS, total score*			
Mean (s.d.)	78.9 (15.4)	56	103
Levodopa dose equivalents (s.d.)	557 (477)	0	1,600

Abbreviations: PD, Parkinson’s disease; UPDRS, Unified PD Rating Scale.

**Table 2:** Change in brain glutathione levels (as GSH/Cr peak ratios) relative to baseline after 200 mg nasally administered GSH

	*Glutathione level (GSH/Cr peak ratio)*				
*Time post dose (min) as midpoint of scan (s.e.m.)*	*Subjects (*n=*15)*	*Mean (s.e.m.)*	*Min*	*Max*	*Mean difference relative to baseline (s.e.m.)*
0 (baseline)	15^a^	0.0170 (0.0046)	0.0035	0.0620	
7.5 (0.0)	15	0.0259 (0.0039)	0.0080	0.0580	0.00890 (0.00507)
19.9 (0.17)	14	0.0364 (0.0057)	0.0120	0.0760	0.0201 (0.00585)
32.0 (0.17)	15	0.0385 (0.0053)	0.0130	0.0810	0.0215 (0.00532)
44.7 (0.22)	9	0.0457 (0.012)	0.0130	0.114	0.0340 (0.0135)

Abbreviations: GSH, glutathione; Cr, creatine.

a For the baseline scans where the GSH peak was undetectable, a GSH/Cr value (lowest measured value/2) was substituted, as described in the Materials and Methods section.

For six subjects, baseline GSH levels were undetectable, and one post-dose spectrum from one subject was omitted owing to poor data quality.
